# Spectrum of head and neck cancer in children

**DOI:** 10.4103/0971-9261.59601

**Published:** 2009

**Authors:** Subhabrata Sengupta, Ranabir Pal, Somnath Saha, S. P. Bera, Indranil Pal, Isha Preet Tuli

**Affiliations:** Department of Oto-Rhino-Laryngology, Sikkim-Manipal Institute of Medical Sciences (SMIMS) and Central Referral Hospital (CRH), 5th Mile Tadong, Gangtok, Sikkim-737 102, India; 1 Department of Community Medicine, Sikkim-Manipal Institute of Medical Sciences (SMIMS) and Central Referral Hospital (CRH), 5th Mile Tadong, Gangtok, Sikkim-737 102, India; 2 Department of Oto-Rhino-Laryngology, Bankura Sammilani Medical College, Bankura, West Bengal, India; 3 Department of Oto-Rhino-Laryngology, Medical College, Calcutta, India

**Keywords:** Head and neck tumor, lymphoma, rhabdomyosarcoma

## Abstract

**Aim:**

To assess the incidence of different head and neck cancers in pediatric age group in a referral hospital.

**Methods:**

In this prospective study, children below the age of 12 years underwent a thorough clinical, ENT examination and the diagnosis was conformed histologically in all the cases.

**Results:**

Fifty-three were diagnosed to be suffering from different head and neck neoplasms among 21,216 children (0.25%). Male-to-female sex distribution was 1.78:1. The lymphomas were the most common (43.39%) followed by the rhabdomyosarcoma (20.75%) and the nasopharyngeal carcinoma (15.09%). Of the lymphomas, the non-Hodgkin's lymphoma was predominant (26.41%). Other lesions were thyroid carcinomas and mucoepidermoid carcinoma of parotid.

**Conclusions:**

Malignancy should always be considered in the differential diagnosis of masses in the head and neck region in children.

## INTRODUCTION

The majority of head and neck masses in children are inflammatory in nature, but other etiologies include congenital, inflammatory, and neoplastic lesions. Persistent adenopathy raises more concerns, especially enlarged lymph nodes within the posterior triangle or supraclavicular space, nodes that are painless, firm, and not mobile, or a single dominant node that persists for more than 6 weeks should all heighten concern for malignancy.[1] Neoplasms of the head and neck region account for approximately 5% of all childhood malignancies.[2]

## MATERIALS AND METHODS

This is a prospective study of patients attending the otorhinolaryngology outpatient department at a tertiary care teaching hospital at Calcutta from 2001 to 2004. Institutional ethical committee approved the study. The main outcome measures were the age-sex distribution and diagnosis of pediatric head and neck cancer. Primarily intracranial and intraorbital cancers were excluded from the study. After clinical assessment, a fine needle aspiration cytology and biopsy for histopathological study was done in all the cases for a confirmed pathological diagnosis. Endoscopic procedures, investigations like X-ray, computer-aided tomographic (CAT) scans, and magnetic resonance imaging of the head and neck region were also done as required to arrive at the diagnosis. All the histologically confirmed cases were followed by interventions according to standard clinical protocols.

## RESULTS

Total number of patients was 137,028 during the study period of 3 years. Of them 21,216 were less than 12 years of age. Among them, 53 were diagnosed with head and neck cancer. Lymphomas were the most common (43.39%) malignant lesion followed by the rhabdomyosarcoma (20.75%) and then the nasopharyngeal carcinoma (15.09%). Of the lymphomas, the non-Hodgkin's lymphoma was predominant (26.41%). Other malignancies like thyroid carcinomas and mucoepidermoid carcinoma of parotid were less frequently encountered.

The malignant lesions were found predominantly above 5 years of age (69.81%), maximum occurring in the age group of 10–12 years (47.17%), followed by the cases in the age group of 6–9 years (22.64%), 2–5 years (18.87%), and only 11.32% were in the 0–1 year age group. The overall sex ratio was 1.78:1 in favor of the males except in thyroid carcinoma and neuroblastoma where the ratio was equal. Eosinophilic granuloma was found only in two male children [Table 1]. Table 2 depicts the comparison of different series of pediatric head and neck cancers.

**Table 1:** Distribution of Different Categories of Childhood Cancers

Histopathological diagnoses	0–1 yrs		2–5 yrs		6–9 yrs		10–12 yrs		Frequency		Male: Female	Total	Percentage
								
	M	F	M	F	M	F	M	F	M	F
Non-Hodgkin Lymphoma	1	1	2	1	2	1	4	2	9	5	1.8:1	14	26.41
Hodgkin lymphoma	0	0	1	1	2	1	3	1	6	3	2:1	9	16.98
Rhabdomyosarcoma	1	0	2	1	2	1	2	2	7	4	1.75:1	11	20.75
Nasopharyngeal carcinoma	0	0	1	0	1	1	3	2	5	3	1.67:1	8	15.09
Thyroid carcinoma	0	0	0	0	0	0	2	2	2	2	1:1	4	7.55
Salivary Carcinoma	0	0	0	0	0	1	2	0	2	1	2:1	3	5.66
Neuroblastoma	1	1	0	0	0	0	0	0	1	1	1:1	2	3.77
Eosinophilic granuloma	1	0	1	0	0	0	0	0	2	0	2:0	2	3.77
Total	4	2	7	3	7	5	16	9	34	19	1.79:1	53	100.00
Percentage	11.32		18.87		22.64		47.17						
Male:Female	2:1		2.33:1		1.4:1		2.29:1		1.79:1				

**Table 2:** Comparison of Data from Different Series of Pediatric Head and Neck Cancer

Types	Medical College and Hospitals, Calcutta 2001–2004	Rapidis *et al*[6]	Robinson *et al*[8]	Cunningham *et al*[9]	Albright *et al*[5]
Hodgkin's Lymphoma	9(16.98)	82(26.6)	31(30.7)	85(35.2)	515(16.9)
Non-Hodgkin's Lymphoma	14(26.41)	78(25.3)	23(22.7)	58(24.1)	301(9.9)
Rhabdomyosarcoma	11(20.75)	68(22.1)	26(25.7)	31(12.8)	239(7.8)
Other soft-tissue sarcoma		31(10.1)	5(4.9)	8(3.3)	127(4.2)
Osteogenic sarcoma				1(0.4)	21(0.7)
Chondrosarcoma					13(0.4)
Other skeletal sarcomas					8(0.3)
Ewing's sarcoma				2(0.8)	20(0.7)
Salivary malignancy	3(5.66)	1(0.3)		6(2.4)	160(5.2)
Thyroid malignancy	4(7.55)	12(3.9)	1(0.9)	24(9.9)	652(21.4)
Nasopharyngeal carcinoma	8(15.09)	18(5.8)	1(0.9)	12(4.9)	54(1.8)
Neuroblastoma	2(3.77)	18(5.8)	14(13.8)	12(4.9)	48(1.6)
Retinoblstoma					497(16.3)
Other neural malignancies					161(5.3)
Malignant Melanoma					132(4.3)
Malignant Teratoma				2(0.8)	18(0.6)
Eosinophilic granuloma	2(3.77)				
Other malignancies					84(2.8)
Total	53	308	101	241	3050

Figures in parentheses are in percentage

## DISCUSSION

Incidence of head and neck tumors in children is relatively rare, but they are on the rise. In the United States, approximately 1 in 333 individuals between the ages of 0 and 20 years will be newly diagnosed with cancer each year, affecting a total of nearly 7,500 children under the age of 15 years and another 3,500 adolescents between 15 and 20 years of age. Five percent of all childhood cancers are head and neck malignancies, thereby affecting approximately 550 children every year.[3] Other studies also confirm this ever increasing trend of childhood malignancies. The overall annual incidence of cancer in children under 15 years of age rose from 11.22 cases/100,000 person-years in the time period of 1973–1975, to 14.03 cases/100,000 person-years in 1994–1996—an increase of 25%; an even larger increase in the incidence of pediatric head and neck malignancies. In this subset, the incidence rate increased from 1.10 to 1.49 cases/100,000 person-years in the same timeframe—an increase of 35%.[4]

In our series, lymphomas were the most common (43.39%) followed by rhabdomyosarcoma, nasopharyngeal carcinoma, and others like thyroid carcinomas. Other studies observed between 7% to 60% of lymphoma among pediatric population.[3–10] Rhabdomyosarcoma was our second highest observation, similar to others.[5,7,8,10]

Global literature delineates common pediatric head and neck tumors as lymphoma (59%), rhabdomyosarcoma (13%), thyroid (10%), nasopharyngeal carcinoma (5%), neuroblastoma (5%), non-rhabdomyosarcoma soft-tissue sarcoma (4.5%), salivary gland malignancies (2.5%), and malignant teratomas (1%).[3] In a more recent study from the United States, lesions involving the head and neck were the most common cancer type (27%), followed by neural tumors (23%), thyroid malignancies (21%), and soft-tissue sarcomas (12%). Papillary thyroid carcinoma (18%) was the leading pathologic diagnosis. In the Rapidis study, lymphomas accounted for 52.3% of the malignant neoplasms—the most frequent of the head and neck malignancies followed by rhabdomyosarcoma and other soft-tissue sarcomas.[5]

However, a recent study from Germany shows slightly different results. In this study, the most frequently observed entities, representing primary tumors in the head and neck in children, were soft-tissue sarcomas (0.39/100000), followed by lymphomas (0.09/100000) and thyroid carcinoma (0.07/100000).[11]

In our study population, the malignant lesions were found predominantly above 5 years of age (69.81%), maximum occurring in the age group of 10–12 years (47.17%). The overall sex ratio was 1.78:1 in favor of the males. Other researchers in this field found mean age of 10.4 years and the male:female ratio is 1:1.12. Head and neck cancers were most frequent among teenagers 15–18 years old (39%), followed by children 4 years or younger (27%), 10–14 years (21%), and 5–9 years (13%).[4] The overall sex ratio in favor of the males was comparable with the studies where the male:female ratio were 1.5:1[5] and 1.16:1.[11] However, in the latter study incidence increased in girls with age and exceeded that of boys in the age group between 10 and 14 years.[11]

In a Nigerian study among general population, cancers displayed male predominance, with a gender ratio of 1.8:1; 2.4% of carcinomas were occurring in children. Hematopoietic malignancies constituted 20.4% of head and neck cancers, and comprised mainly lymphomas, which accounted for 19.3% of all head and neck cancers. The most common childhood malignancy was Burkitt's lymphoma, which comprised 28.2% of pediatric head and neck cancers.[12]

The limitation of this study was that we presented an analysis of head and neck cancer in a tertiary center in eastern India. The percentages were apparent among all the children within this referral center during the study period. It would have been better if we could do a population-based study.

## CONCLUSION

Incidence of head and neck tumors in pediatric age group is very rare. It is only 0.25% of pediatric patients attending the ENT department in three calender years. Among the malignant lesions, lymphomas are most frequent. Incidence of non-Hodgkin's lymphomas is more than Hodgkin's disease. Commonest age of presentation of the malignant diseases is above 10 years. There is overall male predominance with a male:female ratio of 1.78:1. Awareness of a potential malignancy and careful follow-up of children with suspicious head and neck cancers is mandatory so that more and more head and neck cancers in children are brought to treatment before it is too late.[13]
